# Retraction: Immunoinformatic strategy for developing multi-epitope subunit vaccine against *Helicobacter pylori*

**DOI:** 10.1371/journal.pone.0323864

**Published:** 2025-05-01

**Authors:** The PLOS One Editors

The *PLOS One* Editors retract this article [1] due to concerns about compromised peer review, authorship, and potential manipulation of the publication process. These concerns call into question the validity of the reported results. We regret that the issues were not identified prior to the article’s publication.

MN, MRK, FR, TAN, TB, and MA did not agree with the retraction. HMR, IB, MRS, RNN, SMAH, and LM either did not respond directly or could not be reached.

## References

[1] Nahian M, Khan MR, Rahman F, Reza HM, Bayil I, Nodee TA, et al. Immunoinformatic strategy for developing multi-epitope subunit vaccine against *Helicobacter pylori*. PLoS ONE. 2025;20(2):e0318750. doi: 10.1371/journal.pone.0318750 39919064 PMC11805379

